# The Connexion between Out-Patients and Annual Subscriptions

**Published:** 1888-06-09

**Authors:** 


### THE CONNEXION BETWEEN OUT-PATIENTS AND ANNUAL SUBSCRIPTIONS.

The principal reasons for a decrease in the annual subscription to the metropolitan, are not altogether owing to a want of appreciation in the public. In this philanthropic age, when feelings and sympathies are more readily convinced than formerly, there must be some lack of trust to cause a decrease in subscriptions to such charities as our hospitals—charities which appeal constantly not only to the eye but to the imagination of any well-disposed person. The true causes of financial failure The Hospitals Association frequently drew attention to, especially in the earlier days of its work. Surely while the out-patients' department—that great blot on the 'scutcheon of the principal hospitals—remains unreformed, there are other causes besides a lack of money to account for the depression in hospital resources. Miss Octavia Hill, in her late evidence before the Lords Committee on out-door relief, stated that she wished the hospitals of London would reform their system, and we cannot wonder that while they remain practically unreformed, and yet ever increase their appeals for funds, the deficit will continue. There is a feeling of distrust and doubt amongst the many subscribers to hospitals, and it will not disappear till the hospitals themselves show some desire to adopt the many suggestions made for their reform, and to set their affairs in order, as other charities are obliged to do in order to meet the spirit of the day. I trust that The Hospitals Association will continue to point out resolutely that, till a spirit of self-help and reform appears, the hospitals will continue to suffer. This seems to me far more needful a subject than the mere vague sympathy which is constantly found. AN HONORARY SECRETARY.

[We hope An Honorary Secretary and many others will attend the meeting for the discussion of the Pay-System on the 20th June, at the Board-room of St. Thomas's Hospital, at 8 p.m.—ED.]

